# Supplementary material for: Epidermal Growth Factor Receptor (EGFR) Gene Polymorphism May be a Modifier for Cadmium Kidney Toxicity
Source: Genes (Basel). 2021 Oct 2;12(10):1573. doi: 10.3390/genes12101573 (PMC8535213; doi:10.3390/genes12101573)
Supplement: Supplementary file 1 [file genes-12-01573-s001.zip › genes-1368492-SI.pdf]

Table S1. SNP in intron of EGFR on TWB array plate.

Number	rsid	type			n			HW※	Functional	MAF[$]
SNP2	rs4947963	CC	TC	TT	231	210	44	0.834	intron	0.310
SNP3	rs11770506	CC	TC	TT	213	218	57	0.921	intron	0.342
SNP4	rs763317	AA	AG	GG	18	183	288	0.072	intron	0.225
SNP5	rs28557040	AA	GA	GG	423	65	1	1.000	intron	0.070
SNP6	rs6956366	CC	GC	GG	312	164	13	0.199	intron	0.195
SNP7	rs17172425	AA	AG	GG	2	54	433	0.425	intron	0.062
SNP8	rs11760406	AA	GA	GG	342	133	12	1.000	intron	0.161
SNP9	rs11971773	AA	AG	GG	2	54	433	0.425	intron	0.062
SNP10	rs10234806	CC	TC	TT	445	42	2	0.106	intron	0.049
SNP11	rs2302534	CC	CT	TT	3	80	401	0.584	intron	0.091
SNP12	rs17172432	CC	CT	TT	2	82	405	0.786	intron	0.089
SNP13	rs17172434	AA	GA	GG	405	82	2	0.786	intron	0.089
SNP14	rs2302535	AA	AC	CC	6	110	373	1.000	intron	0.125
SNP15	rs11979255	AA	AG	GG	2	44	443	0.351	intron	0.050
SNP16	rs11238349	AA	GA	GG	368	115	6	0.694	intron	0.130
SNP17	rs202028278	AA	AG	GG	6	119	363	0.848	intron	0.135
SNP18	rs6958497	CC	CT	TT	2	79	408	0.568	intron	0.087
SNP19	rs917880	CC	TC	TT	345	129	14	0.739	intron	0.160
SNP20	rs11977660	CC	TC	TT	213	218	57	1.000	intron	0.340
SNP21	rs3823585	CC	CG	GG	43	204	241	1.000	intron	0.298
SNP22	rs3735061	AA	AG	GG	19	162	308	0.786	intron	0.207
SNP23	rs6593205	AA	AG	GG	6	69	414	0.122	intron	0.081
SNP24	rs2110290	CC	TC	TT	245	200	43	0.830	intron	0.294
SNP25	rs12671550	CC	CG	GG	43	198	245	0.829	intron	0.294
SNP26	rs17289413	CC	TC	TT	308	163	18	0.681	intron	0.206
SNP27	rs12668175	CC	GT	TT	101	238	150	0.652	intron	0.452
SNP28	rs12667668	AA	AC	CC	45	232	212	0.129	intron	0.328
SNP29	rs13244925	AA	AC	CC	41	232	215	0.052	intron	0.322
SNP30	rs11534100	CC	CT	TT	222	225	42	0.214	intron	0.314
SNP31	rs4947982	AA	GA	GG	187	246	56	0.068	intron	0.365
SNP32	rs13234622	AA	AG	GG	53	252	183	0.016	intron	0.366
SNP34	rs2075110	CC	TC	TT	157	262	70	0.021	intron	0.410
SNP35	rs4947986	AA	GA	GG	159	261	69	0.021	intron	0.407
SNP36	rs13222549	CC	CG	GG	4	85	398	1.000	intron	0.095

※:Hardy-Weinberg equilibrium, $: Minor allele frequency

Table S1. SNP in intron of EGFR on TWB array plate. (continued)

Number	Rsid	type			n			HW※	Functional	MAF$
SNP37	rs11238354	CC	TC	TT	127	262	98	0.106	intron	0.471
SNP38	rs11536635	AA	AG	GG	108	257	124	0.325	intron	0.486
SNP39	rs1468727	CC	CT	TT	108	258	123	0.325	intron	0.486
SNP40	rs11977388	CC	CT	TT	63	249	177	0.088	intron	0.384
SNP41	rs12669749	AA	AC	CC	64	248	175	0.107	intron	0.386
SNP42	rs845551	AA	AG	GG	68	262	156	0.012	intron	0.409
SNP44	rs75529744	AA	AG	GG	2	78	409	0.562	intron	0.084
SNP45	rs10277413	GG	TG	TT	128	264	97	0.088	intron	0.469
SNP46	rs9642393	CC	TC	TT	147	271	69	0.002	intron	0.419
SNP47	rs845555	CC	CT	TT	82	262	145	0.045	intron	0.435
SNP48	rs7795728	CC	CG	GG	13	126	347	0.619	intron	0.160
SNP49	rs7795743	AA	AG	GG	13	126	347	0.867	intron	0.159
SNP50	rs13222385	AA	GA	GG	400	85	4	1.000	intron	0.095
SNP51	rs6970029	GG	GT	TT	13	127	347	0.868	intron	0.160
SNP52	rs845561	CC	TC	TT	303	163	23	1.000	intron	0.216
SNP53	rs2075106	CC	TC	TT	348	127	12	1.000	intron	0.158
SNP54	rs2075108	AA	GA	GG	201	234	54	0.239	intron	0.350
SNP55	rs2740762	-	AC	CC	-	57	432	0.398	intron	0.080
SNP56	rs1404908	CC	TC	TT	407	80	2	0.410	intron	0.088
SNP57	rs2472520	CC	CG	GG	9	133	347	0.609	intron	0.156
SNP58	rs17290629	GG	GT	TT	41	198	248	0.913	intron	0.286
SNP59	rs2293348	CC	TC	TT	383	102	3	0.249	intron	0.110
SNP61	rs2692456	AA	GA	-	421	68	-	0.158	intron	0.070
SNP82	rs2037701	AA	AG	GG	12	144	332	0.527	intron	0.171
SNP83	rs73139030	CC	TC	TT	222	223	43	0.252	intron	0.314
SNP84	rs4947995	AA	AG	GG	115	250	121	0.590	intron	0.495
SNP85	rs11975042	AA	GA	GG	226	211	52	0.839	intron	0.325
SNP86	rs1534130		AG	GG	-	52	437	0.387	intron	0.052
SNP87	rs11982525	CC	CT	TT	48	220	220	0.474	intron	0.322
SNP88	rs11767730	CC	TC	TT	313	150	24	0.271	intron	0.205
SNP89	rs12718950	AA	GA	GG	221	220	48	0.538	intron	0.321

※: Hardy-Weinberg equilibrium, $: Minor allele frequency

Table S2. SNP in NOT intron of EGFR on TWB array plate.

Number	Rsid	type			n			HW※	Functional	MAF$
SNP1	rs6965469	CC	TC	TT	314	163	11	0.042	a	0.190
SNP33	rs2072454	CC	TC	TT	157	264	68	0.010	b	0.408
SNP43	rs10258429	CC	TC	TT	417	68	4	0.536	b	0.079
SNP60	rs2293347	CC	TC	TT	250	197	42	0.743	b	0.288
SNP62	rs884225	CC	TC	CC	130	247	111	0.858	c	0.481
SNP63	rs2280653	AA	GA	GG	252	193	44	0.381	d	0.286
SNP64	rs940810	CC	TC	-	439	50	-	0.626	none	0.050
SNP65	rs7334	AA	CA	CC	309	164	14	0.208	none	0.199
SNP66	rs940807	AA	AC	CC	45	193	250	0.383	none	0.287
SNP67	rs940806	-	AG	GG	-	80	408	0.038	none	0.083
SNP68	rs1107616	AA	GA	GG	316	157	15	0.387	none	0.192
SNP69	rs7809028	AA	AG	GG	42	194	253	0.510	none	0.283
SNP70	rs67031496	AA	GA	GG	387	99	3	0.248	none	0.108
SNP71	rs7783970	AA	GA	GG	147	242	100	0.928	none	0.451
SNP72	rs41324647	AA	GA	GG	227	220	40	0.172	none	0.306
SNP73	rs12535328	CC	CT	TT	5	111	373	0.405	none	0.123
SNP74	rs10233099	CC	TC	TT	224	211	53	0.761	none	0.328
SNP75	rs12718947	CC	TC	TT	325	145	19	0.466	none	0.188
SNP76	rs12670163	AA	GA	GG	417	69	2	1.000	none	0.074
SNP77	rs7809332	CC	CT	TT	73	226	188	0.705	none	0.383
SNP78	rs1525643	AA	AG	GG	19	149	321	0.665	none	0.192
SNP79	rs868254	CC	CG	GG	114	245	128	0.858	none	0.483
SNP80	rs4245566	AA	AG	GG	12	149	327	0.287	none	0.178
SNP92	rs6593214	AA	AT	TT	30	174	281	0.711	none	0.239
SNP93	rs1525642	CC	CT	TT	68	222	199	0.700	none	0.366

※: Hardy-Weinberg equilibrium, $: Minor allele frequency, a : 2KB Upstream Variant

b : Synonymous Variant, c : 3 Prime UTR Variant, d : 500B Downstream Variant

Table S3. EGFR long non-coding downstream RNA (ELDR) on TWB array plate.

Number	Rsid	type			n			HW※	Functional	MAF$
SNP81	rs4947993	CC	CT	TT	57	248	184	0.054	e	0.367
SNP90	rs6948867	AA	AG	GG	32	175	279	0.543	f	0.244
SNP91	rs35891645	CC	TC	TT	282	173	33	0.393	g	0.243

※: Hardy-Weinberg equilibrium, $: Minor allele frequency, e : 500B Downstream Variant

f : Non Coding Transcript Variant, g : 2KB Upstream Variant
